# Hospital and Institutional News

**Published:** 1915-06-19

**Authors:** 


### HOSPITAL AND INSTITUTIONAL NEWS.

### THE KING AND HOSPITAL SUNDAY.

The message which accompanied the King's cheque for £100 towards the Metropolitan Hospital Sunday Fund aptly summed up the burden which the voluntary hospitals are bearing as a result of the war. "I am commanded by the King," wrote Sir F. Ponsonby in forwarding His Majesty's donation, "to send you the enclosed cheque for £100 for the Hospital Sunday Fund, on behalf of which His Majesty notices you are making a special appeal on account of the unusual strain upon all the London hospitals occasioned by the war." Hospital Sunday was observed at practically every place of worship in the capital, and the special offertories held on behalf of the fund were supplemented by a number of voluntary street collectors. Unusual significance will attach to the result as an index of the degree in which the public grasp how the hospitals best represent the demand for "national mobilisation," and we await it with confidence.

### PRECAUTIONS AGAINST POISONOUS GASES: A PARISIAN FORMULA.

Concerning means for combating the effects of poisonous gases used by the Germans, a vast amount of printed matter has appeared in the lay Press. A host of suggestions have been made; but, after all has been said, little of value emerges from these discussions beyond the original proposal to use respirators treated with certain chemical compounds which, in contact with chlorine, are converted into innocuous substances. Some difference of opinion exists with regard to the precise proportions of the chemical compounds that should be used in solutions for treating the respirators, but it would be difficult to improve upon the formula which has been adopted by the Paris Academy of Medicine—namely, hyposulphite of soda, 1,000 grammes; carbonate of soda crystals, 200 grammes; glycerine, 150 grammes; water, 800 grammes. The object of the glycerine is to keep the respirator moist. Inquiries at some of the large London emporiums show that quite a considerable business is being done in respirators, and solutions for moistening them, for use in the event of an air raid. While it is obviously wise to take reasonable precautions to ensure, as far as possible, safety from poisonous gases, it is questionable whether more harm is not being done by nervous apprehension than could be done by an air raid on London.

### THE "M.A.B." AND WOUNDED SOLDIERS.

Pressure has recently been brought to bear on the Metropolitan Asylums Board with a view to their sparing even more of their accommodation than they have already done for wounded soldiers. It must be said that the Board has rendered inestimable service to the War Office not only in setting aside the Brook and Southern Hospitals for wounded soldiers, but in finding accommodation for the large numbers of Belgian refugees who have been sent to London. After carefully considering the whole question it appeared that the only institution at present available for wounded soldiers was the High Wood School, where cases of ophthalmia are treated, and the patients now in that school have been transferred to the White Oak School. The High Wood School has therefore been offered to the Government as a military hospital, and has been officially accepted by the Local Government Board, who have thanked the Board for their offer. The Brook Hospital has been designated by the military authorities as "The Brook War Hospital," and the Southern (Lower) Hospital as "The Dartford War Hospital."

### THE MAUDSLEY WAR HOSPITAL.

An intimation having been received from the Board of Control that the Army Council would be glad to obtain, for the treatment of soldiers suffering from nervous disorders, the use of the Maudsley Hospital buildings and of Osnabruck House (the adjacent property which has been acquired for use as a nurses' home for the hospital), an offer of the use of the premises, free of charge, has been made. The understanding is that the War Office will pay all outgoings in connection with the hospital if it is so used. Moreover, since the hospital buildings are not yet completed, and some variations in the work which remains to be done under contract may be necessary if the buildings are to be used temporarily for soldiers, the War Office are also to agree to refund any extra cost to which the Council may be put in completing the buildings at a later date. The hospital, if used by the Army Council, will be attached to the 4th London General Hospital, and the County Council will not be associated with its administration. They have asked, however, that, if possible, the name of Dr. Maudsley shall continue to be associated with the institution as a war hospital.

### A GERMAN MATRON: APPEAL TO THE SECRETARY OF STATE.

Since our last issue went to press, Dr. Leonard Raby, surgeon to the Devizes Cottage Hospital, has sent a letter to the local Press in which he gives certain important facts that may well prove decisive in the regrettable discussion as to whether or not the services of the present matron, a German by birth, should be retained. As we have already recorded, the action of the committee in retaining the lady on their staff has led to some criticism, and much local controversy, while the medical staff have defended the committee and the matron in a letter, from which we quoted. In the communication of Dr. Raby, however, which appeared on June 10, he says: "Immediately war broke out, a special meeting of the committee was held to consider her (the matron's) position, and being unanimously convinced of her loyalty, and knowing that for seventeen years she had made her home in England . . . the committee determined that her services should be retained. Upon receiving a letter from Mr. Gwatkin dated October 14, 1914, declining to subscribe any more until the matron was removed, it was decided to consult the Secretary of State. . . . The Secretary of State replied on November 16, 1914: 'He does not think that the committee need take any action in the matter.'" This should be sufficient to dispose of the controversy; and had these instructions from headquarters been made public as soon as they were received, a very regrettable controversy would have been avoided.

### THE DELAY IN NATURALISATION.

It only remains to add that Dr. Raby states that Miss Rettberg obtained her naturalisation papers on November 27, 1911, when at Tamworth Hospital. Owing to her having been engaged in private nursing, she found it difficult, he adds, to give the required account of all the places she had lived at and the dates for five consecutive years. In June 1914, therefore, when she became better able to give an uninterrupted record at Tamworth and Devizes, she sent the papers to a solicitor to be completed. By "an unavoidable delay" they were not sent to the Home Secretary till August. War had then been declared, and the matter was held over. This completes Dr. Raby's account of the matter.

### THE SECOND MILITARY HOSPITAL AT CARDIFF OPENED.

Cardiff has become a city to which two separate military hospitals are attached. Since August last the 3rd Western General Hospital has been in full working order, and on June 9 the City Mental Hospital was also formally opened under the title of the Welsh Metropolitan War Hospital. A batch of wounded has already arrived. All the staff have been taken over by the War Office at their old rates of remuneration, and the buildings and their equipment, which have cost over £300,000, are being given rent free as a patriotic gift on the part of the city. An outlay of over £7,000 has already been incurred by the War Office in necessary alterations and additions, which include an operating theatre, a new pack store, a large dispensary annexe, and additional bedsteads and furniture to accommodate 900 men. The total number of mental patients for which the institution had been formerly equipped was 750. The following constitute the official staff: Lieut.-Colonel Edwin Goodall, M.D., F.R.C.P., administrator; Major E. B. C. White, M.R.C.S., L.R.C.P., registrar; Major H. D. Harrison, M.B., F.R.C.S., chief resident surgeon; Major C. H. G. Ramsbottom, M.D., chief resident physician; Dr. R. J. Waugh, M.B., resident physician; Mr. C. N. Williams, resident surgeon; R. V. Stanford, M.Sc., resident chemist; J. D. Fry, B.Sc., resident radiographer; W. Kittow, dentist; Councillor Edward England, transport officer; J. D. Morgan, clerk and steward; Miss F. E. Raynes, matron; C. H. Humber, orderly inspector. There are 12 visiting surgeons, 110 nurses, and about 90 orderlies. Other appointments on the medical staff are to be made, including that of pathologist and bacteriologist.

### CLERGYMEN AS ARMY DRIVERS.

One of the remarkable effects of the war has been to reveal our clergy as among the most pugnacious members of the community. Some have enlisted, many more have chafed at the virtual prohibition contained in the Archbishop's recent pronouncement, and large numbers are seeking various noncombatant posts. A good example of the way in which their services are being utilised in transport is contained in the announcement that the twenty-five ambulances which the Church Army is presenting for Red Cross work at the Front are being driven by clergymen; and indeed it is perhaps fitting that the Church Army and its officers or clerical colleagues should play an active part in the field. Twelve ambulances and a kitchen car were dedicated by the Archbishop of Canterbury last week. The kitchen car, which may be compared with the one which was described and illustrated in The Hospital's Special Number of April 17, p. 56, can provide 2,600 cups of tea, and its appliances include a tube for pumping water out of any river, fitted with a filter which works in combination with the pump. It is interesting to add that each ambulance car is provided with a paten and chalice, thus, as it were, setting the seal of Christian and clerical work on that modern embodiment of the Good Samaritan which is what a motor ambulance actually is. With the clergy as drivers these ambulances provide the most practical channel, apart from chaplain's work, for clerical enthusiasm.

### THE WAR AND THE EPILEPTIC COLONY.

Dr. M. A. Collins, the medical superintendent at the Epileptic Colony of the London County Council, and Dr. A. A. W. Petrie, the assistant medical officer, have been granted permission by the London Mental Hospitals Committee to accept commissions in the Royal Army Medical Corps, and temporary arrangements are being made for the medical administration of the colony.

### NEW DIETARIES IN POOR-LAW INSTITUTIONS.

Owing to the price of meat a change of diet has been decided on by the Halifax Board of Guardians in their workhouse and also in the infirmary. The changes in the dietary of the former are given as follows, and will have a certain historical value when the effects of the war on institutional life come to be considered. In place of the boiled beef and potatoes served out for dinners for all classes of men and women on Thursdays there is substituted: For men, 8 oz. of bread, 3 oz. of cheese, and one pint of coffee; for women, 6 oz. of bread, 2 oz. of cheese, and one pint of coffee. On Saturdays the customary Irish stew and boiled mutton are being replaced by one and a half pints of pea or haricot soup with bread, on the same scale as above for the two sexes. Presumably, although diet changes are being made in the hospital, such changes will be, at all events in individual cases, subject to the consent of the medical superintendent, who must retain his power to order additional diet for any patient who, in his judgment, requires special or more nourishing food. A point is being made that the officers should also be subject to modifications of diet, and, at all events in other Unions, fish dinners on one or more days a week are a favourite amendment. At Keighley, for instance, the new dietary tables introduced by the Board are expected to reduce the cost in food per head from 5s. 3d. to 4s. In two months' time the committee is to ascertain the extent of the reductions, and in this way it is to be hoped that false economy will be avoided.

### A NEW WOMAN'S POST IN THE PUBLIC HEALTH DEPARTMENT.

About a year ago the London County Council decided to appoint a woman inspector in the public health department in connection with the administration of the Mental Deficiency Act at a commencing salary of £150 a year, and applications for the post were invited by public advertisement. Forty applications have been received, and five candidates were interviewed by the Mental Hospitals Committee in January last. It is now proposed to appoint to the position Miss Annie Adeline Curran, who has been acting as woman inspector temporarily since June 1914. Miss Curran is forty-two years of age. Her salary will rise by annual increments of £10 a year to £200.

### THE LACK OF MENTAL HOSPITAL BEDS.

In ordinary times the problem of finding accommodation for London's mental cases in its mental hospitals is sufficiently serious, but in time of war it is doubly so. Overcrowding of the other London County mental hospitals, consequent upon the arrangements made for the removal of the patients from Horton Mental Hospital, to allow of its use as a war hospital, and the difficulty which is being found in providing an adequate male staff for the care of the patients at the mental hospitals in consequence of the enlistment of attendants, temporary substitutes for whom must not be eligible for military service, have led the Mental Hospitals Committee to make a new regulation. This is to the effect "that during the use of Horton as a war hospital, beds which become vacant at the London County mental hospitals shall be reserved for cases of acute insanity, or cases of chronic insanity, which are urgently in need of asylum treatment, and cannot be dealt with otherwise; and that, as respects the admission of cases not within these classes, the London County mental hospitals shall be deemed to be full unless the committee decide to fill any beds so reserved with patients not of the classes for which they are reserved."

### WEST END HOSPITAL AND THE WOUNDED.

The senior surgeon to the hospital, Mr. C. A. Ballance, M.V.O., M.S., started for Malta at the end of last month, having been asked to go out immediately as consulting surgeon to the Army. This week one of the physicians, Dr. Purves Stewart, follows to the Mediterranean, having been appointed a consulting physician to H.M. Forces. Notwithstanding staff difficulties, however, the work for civilians, adults and children, is being carried on on the same scale as at ordinary times; and in addition a number of beds has been opened specially for nerve-injured and nerve-wrecked soldiers, of whom as many as twenty-one have been in the hospital at one time. The hospital has, unfortunately, no garden of its own; but the loss is being made up through the kindness of some of the owners of the beautiful gardens in the locality, who are permitting the men to have access to them under proper hospital escort. Owing to the extra number of beds it has been found necessary to obtain assistance for the house physician, and Mr. G. C. Kusumbeker, M.R.C.S., has been appointed assistant house physician. Mr. Philip Figdor, M.B., has been appointed a clinical assistant. These facts show that while existing nerve hospitals have little spare accommodation, yet they are able to take part in caring for soldiers suffering from nervous disorders.

### FIVE HUNDRED CASES FOR TEN GUINEAS.

An appeal was made to the Wandsworth Board of Guardians to increase their annual subscription of £10 10s. to the Victoria Hospital for Children, but the appeal fell on an invincible Board determined not to increase their donation to an institution that was of inestimable value to them. It was ascertained that in the last year no fewer than 518 children were sent to the hospital by the Guardians. Miss E. Hill thought it was the duty of the Guardians to do everything possible in the interests of the child life of the borough, and desired a revision of the proposal to grant the hospital only ten guineas. Mr. J. Norman said the Guardians took no more from the hospital than what they were entitled to, and if other children were sent, letters other than those granted to the Board had to be found ere the children were admitted. Even to the Bolingbroke Hospital, to which the Board is particularly indebted, a subscription of only £10 10s. is given, so that it was apparent that the Board are far from generous in their subscriptions. Miss Hill's attempt to get an increased donation was not successful, the Guardians deciding that £10 10s. yearly was sufficient to cover the cost of the treatment of 518 children.

### THE NEW SANATORIA FOR LONDON CONSUMPTIVES.

Although the present time is hardly favourable for the carrying out of new building schemes, it appears probable that one at least of the three new sanatoria for the accommodation of Metropolitan consumptives will be completed this year. A sub-committee of the Metropolitan Asylums Board approved some six months ago the plans drawn up by Mr. E. T. Hall, F.R.I.B.A., for the erection of three sanatoria—namely, one for 232 women at Hyde Style, near Godalming, at a cost of £42,138; one for 168 men at Felbridge, near East Grinstead, to cost £29,000, and another, for 175 men, at Ellisfield, near Basingstoke, to cost £30,900. The consent of the Local Government Board was obtained for the first one, at Godalming, and considerable progress has been made with the construction of the building. It is understood that the others will be proceeded with also as soon as possible. At present the Down Schools, formerly used for children with ringworm, and the Northern Hospital for fever convalescents, at Winchmore Hill, are being used for the accommodation of tuberculous cases sent by the London Insurance Committee. Cases coming under the London County Council scheme, and a few sent by the Middlesex County Council, are also to be found at these institutions. At the Northern Hospital there are about 220 tuberculous cases in residence every day, and this means that the accommodation for convalescing fever patients is reduced materially, for consumptives take up more room than fever children, who form the bulk of the normal patients at Winchmore Hill. Hence it would seem excusable to push on the construction of the sanatoria in order to set free as many fever beds as possible for eventualities next winter. But it may be expected that the friends of the patients will hardly welcome the prospect of having to make longer journeys when they obtain visiting permits.

### YORK HOSPITAL AND ITS FINANCES.

The quarterly court of the governors of York County Hospital, which was held last week, was faced with an unusual position, since there was presented to it the annual report held over, as we recorded at the time, from the preceding annual meeting. The delay in its presentation, due to the prolonged ill-health of Mr. F. Neden, the secretary, has come to an end, and he has happily recovered. But the financial situation has certainly not improved. The accumulated deficit amounts to practically £4,000, of which some £1,800 is the overdraft for last year alone. Meantime the number of in-patients is increasing (150 more were treated last year), and no plan of financial campaign has yet been seriously undertaken. It is true that a committee was appointed last year to make suggestions and formulate a policy, but its brief labours were abandoned on the outbreak of war, and the only inspiriting fact is that the receipts last year showed some improvement, to which the York workpeople's hospital fund and the house-to-house and workshop collections contributed. We believe, as we have said before, that a fact of this kind is an indication of the lines which a new forward financial policy should follow. We also believe that the war, far from being made an excuse for failure, should provide a powerful means of enlisting public support in York, if only the County Hospital's authorities will set to work in an energetic and intelligent way. The presence of soldiers in our voluntary hospitals has affected the public imagination more than any recent work that the hospitals have undertaken, and only good management is required to use the public desire to benefit the wounded to cover also the needs of the institutions in which they are being restored to health. Soldier beds can be supported, as at Norwich; extended grants from the working men be gained as at Cardiff, as local circumstances may decide. The opportunity is there, but brains and experience are required to make use of it.

### COTTAGE HOMES FOR POOR-LAW CHILDREN.

The new cottage homes for the children under the care of the Fylde Poor-Law Guardians were opened last week. Forming three pairs of semi-detached cottages, built on the site of the old workhouse at Kirkham, the new homes have been built from the designs of Mr. Fred. Harrison, the architect, at a cost of £10,000. The chairman of the building committee, Mr. W. N. Bradley, in asking the Guardians to accept the homes, declared that the Fylde Union was the richest in England and also the most economically managed. Mr. W. Hodgson, the chairman of the Board, said that the cost seemed great, but that the homes formed an experiment which the Guardians could well afford to carry out. But at the same time it was not so much the character of the building in which the children were housed upon which their future depended, as upon the nature of those placed in charge of them. Other speakers pointed out that every attempt was being made to make the homes as much like ordinary homes as possible. We all appreciate what this phrase means, but at the same time the cry is so often raised that the private individual home is the only home worth having that it is well sometimes to remember that individual homes are as much liable to abuse as those under public control, and that Poor-Law children run more risk than most of us as to the sort of home in which circumstances may cast them. These cottage homes at Kirkham accommodate eighty-four children, and the experiment will be watched with interest.

### ANOMALIES IN THE POOR-LAW SERVICE.

Reference was made in the columns of The Hospital of May 8 to the loss the Southwark Board of Guardians sustained when the assistant medical officer at their infirmary transferred his services to the neighbouring infirmary at Camberwell because of the enhanced salary offered there. The Southwark Guardians, with a desire to obtain the services of as good a man as the one who had left, and to safeguard themselves from a similar loss in future, applied to the Local Government Board for permission to grant a commencing salary of £250 per year to the assistant medical officer, rising by annual increments of £25 to a maximum of £350 per annum, thus in the end giving the doctor the same salary as is given at Camberwell. The Local Government Board, however, have declined to sanction this, but instead have decided that the salary shall be £250 per annum at the commencement, rising at the end of the year to £275 only. It is evident that steps will have to be taken to remedy anomalies like this, which, unfortunately, are common in the services of the Metropolitan Boards of Guardians relating to the medical staffs.

### ANOTHER REBUFF FOR RECOGNITION OF SERVICES.

The Local Government Board have once again declined to recognise the services of Dr. Keats, medical superintendent of the Camberwell Infirmary. Several applications from the local Board of Guardians were made for permission to pay Dr. Keats an extra £50 a year, but all were refused by the authorities. Then the expedient was devised by the Guardians of allotting to Dr. Keats £50 a year in lieu of rations, or the alternative of permitting him to draw rations from the infirmary stores to the same value, but the Local Government Board have again refused to sanction this. From these various rebuffs it would appear as if the Local Government Board were satisfied that the medical superintendent of Camberwell Infirmary was receiving a salary which they considered adequate. The Guardians have, therefore, to decide whether to allow the matter to drop for the present, and to take it up in a year's time, rather than press their views on a Board which has again and again shown its opinion by refusing any further addition to the salary either directly or by way of emoluments.

### THE MEDICAL STAFF AT EDMONTON MILITARY HOSPITAL.

The appointments to the various positions on the medical and surgical staffs at this hospital have now been made. Lieut.-Colonel Mort, F.R.C.S.E., is the officer in command, with Major Batchelor Taylor as registrar, Major H. M. Rashbrooke as senior resident physician, and Major D. M. Hughes as senior resident surgeon. Dr. Metcalf is radiographer, and Drs. Todrick and Gurney Lessey are the assistant medical officers. All the foregoing will be paid by the War Office, except Lieut.-Colonel Mort, who will be remunerated by the Edmonton Guardians. In addition, the following will act as honorary medical officers: Drs. C. Kirkhope, G. Plaister, and Gurney Thompson. It is proposed to allot the rooms in the administrative block formerly used by the nurses to the medical staff, arrangements having been made with the Edmonton Council whereby Rymmer Park House will be available for the accommodation of forty nurses. The new entrance in Silver Street will be used by the staff and visitors to the hospital, thus avoiding any suggestion of "pauper" taint which might be associated with the use of the workhouse entrance.

### VERSES—AND A PIANO.

Some months ago Miss Augusta Squires, a Leicester authoress, conceived the idea of providing a piano for the Children's Hospital of her town by the sale of her verses, "The Queen Alexandra Rose." With the aid of her friends she soon found herself in possession of £25. With the co-operation of the Mayoress a piano was purchased, and at a recent meeting of the committee of the Leicester Royal Infirmary the Mayor and Mayoress, Alderman and Mrs. North, formally handed over to the institution, on Miss Squires' behalf, her gift, which bore the following inscription: "This piano was presented by Miss Augusta Squires from the sale of her poem, 'The Queen Alexandra Rose,' 1915." We are certain that Miss Squires, whose health is not robust, derived great satisfaction and pleasure from the accomplishment of her self-imposed task, and we record the distinct service she has rendered as an encouragement to the legion of willing friends who, unable of themselves to render monetary service, can in association with others render great assistance to the hospitals if their efforts are well directed.

### A CURIOUS OFFER.

Among the curious offers of gifts received periodically by managers of voluntary hospitals must be reckoned one contained in a letter read at a committee meeting of the Stockport Infirmary last week. A lady, writing from Western Australia, stated that she had heard that a diamond was able to absorb radium emanations if placed in proximity to it. If such were indeed the case she offered to send a diamond from her ring, which she valued at ten pounds sterling, to be used for this purpose at Stockport Infirmary. The Secretary of the institution, Major Tyler, reported that the matter had been submitted to the medical staff of the hospital, who had expressed the opinion that the lady had been misinformed. Diamond rings, however, have a value even if the stone itself stores nothing but the brightness which it reflects. Perhaps the nature of this value will occur to the lady, who may then carry out her original offer.

### THE BRITISH PHARMACEUTICAL CONFERENCE.

In common with all public bodies of the kind, the British Pharmaceutical Conference is feeling the effects of the war. Many of its most prominent members are, in fact, directly or indirectly engaged in the prosecution of the war. For instance, the President, Captain E. S. Peek, is the officer commanding the depôt of the 1st Cambridgeshire Regiment; while others have joined the Colours in one capacity or another, and still others are occupied with war work of one kind or another. Consequently, the Executive Committee has decided that the annual meeting this year shall be of a formal business character. The social side of the meeting is to be abandoned entirely, and there will be no meeting of the Science and Practice Sections. The formal annual meeting will be held in London on July 14. Under normal circumstances the Conference would have met at Scarborough, and no doubt the social programme would have been of a most attractive character. For the time being, however, public social gatherings are out of the question. The Conference numbers among its most active members hospital pharmacists whose time is so fully occupied that they cannot be expected to contribute original papers at present.

### THE SECRETARYSHIP OF THE HAMPSTEAD GENERAL HOSPITAL.

Shortly after we go to press this week a special meeting of the Council is being held to make the final decision among the selected candidates for the post of secretary to the Hampstead General Hospital. As our readers will recall, on the late secretary, Mr. A. E. Thomas, who was killed in action, leaving for military duties, Mr. C. W. Thies for a time acted as honorary secretary. The vacancy was advertised, and considerable comment was caused by the appointment, notwithstanding the terms of the advertisement, of a gentleman who had had virtually no hospital experience. The successful candidate, Mr. J. A. Kyle, who had been actively associated with the Boy Scout movement, has now, however, left to take up military duties, we understand, in the transport service. It became necessary again to advertise the vacancy, and the results of the advertisement proved so overwhelming that we were asked to publish a statement in which candidates were requested to excuse delay in the acknowledgment of their applications, on the ground that the number of these was exceptionally great. This result is in instructive contrast to the difficulty created by the war in securing hospital medical officers and nurses. A sifting process brought the number of selected candidates to ten, who were again reduced to three, and it is out of these three that the selection will be known by the time that this issue is published. Hospital men are waiting to see whether the authorities will maintain their recent policy of seeking a secretary from outside the ranks of experienced institutional officers, or whether, on this occasion, they will prefer a man of hospital training and experience.

### DUTY-FREE SPIRIT FOR HOSPITALS: TEXT OF THE GOVERNMENT PROPOSALS.

An amendment of supreme importance to hospitals has been proposed by the Chancellor of the Exchequer in Committee on the Finance Bill. The amendment seeks to authorise the use of duty-free spirits in hospitals in the preparation of tinctures, etc., and the proposal is similar to one which was made in a recent article in The Hospital. At the time of writing the fate of this amendment was not known, but it is significant that it has been proposed by the Government. The full text of the amendment is as follows:—(1) Section 8 of the Finance Act, 1902 (which gives power to authorise the use of spirits without payment of duty in arts and manufactures), shall apply to the preparation in a public hospital of tinctures and other articles to be used for medical purposes in the hospital as it applies to arts or manufactures in which the use of spirits is required. (2) If the treasurer or other responsible officer of a public hospital shows, to the satisfaction of the Commissioners of Customs and Excise, that any tinctures or other articles which contain spirit, or in the preparation or manufacture of which spirits were used, have within the preceding six months been used for medical purposes in the hospital, he shall be entitled to obtain from the Commissioners repayment of any duty paid in respect of the spirits contained in or used in the preparation or manufacture of the said tinctures or other articles. If any person, for the purpose of obtaining any repayment under the foregoing provision, knowingly makes any false statement or false representation, he shall be liable, on summary conviction, to imprisonment, with or without hard labour, for a term not exceeding six months. (3) For the purpose of the foregoing provision the expression "public hospital" means a hospital supported by any public authority, or wholly or partly out of any public or charitable funds, or by voluntary subscriptions. The saving which hospitals will effect as a result of this provision will, in the aggregate, be very substantial. There is every reason to regard this matter with great satisfaction.

### THIS WEEK'S DRUG MARKET.

There is no abatement of activity in those departments of the market concerned with the supply of drugs for the treatment of wounded soldiers. Makers of morphine, codeine, caffeine, bromides, bismuth preparations, the salts of mercury, etc., are kept constantly busy in their endeavour to meet the colossal demand. As has been the case for many months past, the general tendency of prices is in an upward direction. Nux vomica is getting scarcer and dearer, and it is not improbable that quotations for strychnine will again be advanced. The salicylates still have an upward tendency in price, and the hope that values would be reduced as a consequence of the receipt of larger supplies has almost been abandoned. It seems that in America, from which country further supplies had been expected, salicylic acid and salicylate of soda have advanced in value to a remarkable extent as a result of the rapid and phenomenal rise in the price of carbolic acid—the raw material required for the manufacture of salicylates. Carbolic acid is much dearer in America than it is here, the reason being that such large quantities are required for the production of explosives. There is little material change in the position of Italian produce, but tartaric and citric acids and cream of tartar continue to tend upwards in price. Permanganate of potash is again dearer. Cod-liver oil is unchanged. Sodium hyposulphite is again dearer. Balsam of Peru fetches very high prices. Sulphate of soda is steadily advancing in price. Phenazone and hexamine are dearer. At the recent public auction of drugs held in Mincing Lane, Cape aloes, cardamoms, and buchu leaves inclined lower in price. High prices were paid for senna leaves, and the value of honey was well maintained.

### TO OUR READERS.

Contributions are specially invited from any of our readers to these columns. They should deal with topical subjects and news. They must be authenticated for the information of the Editor only. The minimum payment if published is 5s. There is no hard-and-fast rule as to space, but notes of about twenty lines in length are preferred.

